# GNormPlus: An Integrative Approach for Tagging Genes, Gene Families, and Protein Domains

**DOI:** 10.1155/2015/918710

**Published:** 2015-08-25

**Authors:** Chih-Hsuan Wei, Hung-Yu Kao, Zhiyong Lu

**Affiliations:** ^1^National Center for Biotechnology Information (NCBI), 8600 Rockville Pike, Bethesda, MD 20894, USA; ^2^Department of Computer Science and Information Engineering, National Cheng Kung University, Tainan 701, Taiwan

## Abstract

The automatic recognition of gene names and their associated database identifiers from biomedical text has been widely studied in recent years, as these tasks play an important role in many downstream text-mining applications. Despite significant previous research, only a small number of tools are publicly available and these tools are typically restricted to detecting only mention level gene names or only document level gene identifiers. In this work, we report GNormPlus: an end-to-end and open source system that handles both gene mention and identifier detection. We created a new corpus of 694 PubMed articles to support our development of GNormPlus, containing manual annotations for not only gene names and their identifiers, but also closely related concepts useful for gene name disambiguation, such as gene families and protein domains. GNormPlus integrates several advanced text-mining techniques, including SimConcept for resolving composite gene names. As a result, GNormPlus compares favorably to other state-of-the-art methods when evaluated on two widely used public benchmarking datasets, achieving 86.7% F1-score on the BioCreative II Gene Normalization task dataset and 50.1% F1-score on the BioCreative III Gene Normalization task dataset. The GNormPlus source code and its annotated corpus are freely available, and the results of applying GNormPlus to the entire PubMed are freely accessible through our web-based tool PubTator.

## 1. Introduction

With the rapid growth of biomedical literature, text-mining or biomedical natural language processing (BioNLP) becomes increasingly important for today's biomedical research [1–6]. BioNLP holds the promise to have computers to read the vast amount of the literature and extract key knowledge about specific topics, such as protein-protein/drug-drug interactions [7–11], protein functions and transport [12, 13], and genetic mutations [14–16]. To accomplish that, the first BioNLP task is often known as named entity recognition (NER): to automatically identify the names of biological entities (e.g., gene/protein) from unstructured texts [17]. Given the central role of gene/proteins in the biomedical research [18], the automatic recognition of gene (note that we use gene and protein interchangeably in this paper) names has received much more attention by the BioNLP researchers [19–26] than other entities such as diseases (e.g., DNorm [27]) and chemicals (e.g., tmChem [28]).

Despite many attempts in the past, the gene NER task remains challenging due to both language variation and ambiguity. First, the same gene is often described in multiple different ways by the authors including the orthographical variation (e.g., “ESR1” and “ESR-1”), morphological variation (e.g., “GHF-1 transcriptional factor” and “GHF-1 transcription factor”), variation with abbreviation (e.g., “estrogen receptor alpha (ER*α*)”), and composition mentions (e.g., “BRCA1/2” and “SMADs 1, 5, and 8”). With respect to ambiguity, the first challenge is multispecies (orthologous) gene ambiguity. That is, the same gene name can indicate different concept identifiers depending on its associated organism information (e.g., erbb2 can be either a human gene or mouse gene name). The second ambiguity arises because different genes can share the same name. For example, “AP-1” can refer to either “jun proto-oncogene” (Entrez Gene: 3725) or “FBJ murine osteosarcoma viral oncogene homolog” (Entrez Gene: 2353).

To advance the state of the art in NER, a number of community-wide shared tasks have been organized [29–31] (see Huang and Lu, 2015 [32], for a complete list). In particular, the Critical Assessment of Information Extraction Systems in Biology (BioCreative) has repeatedly organized both gene mention (GM) and gene normalization (GN) tasks where the former task involves finding the occurrence (i.e., string offsets) of gene names in text while the latter typically asks for returning gene concept identifiers per document. In BioCreative I [33] and BioCreative II [7], the GM tasks focused on four species (e.g., human, fly, mouse, and yeast) gene mentions. The best results obtained in the challenges are 83.2% of *F*-measure in BC I GM task [33] and 88.22% in BC II GM task [34]. In BioCreative II, the GN task was introduced which asked participants to return human gene/protein concept identifiers given target articles. The best performance in this task was 81.0% *F*-measure [7]. In BioCreative III, the GN task was reintroduced with the additional challenges of dealing with full text and multiple species. As a result, the best performance is lower (46.56% in *F*-measure [19]).

**Figure 1:**
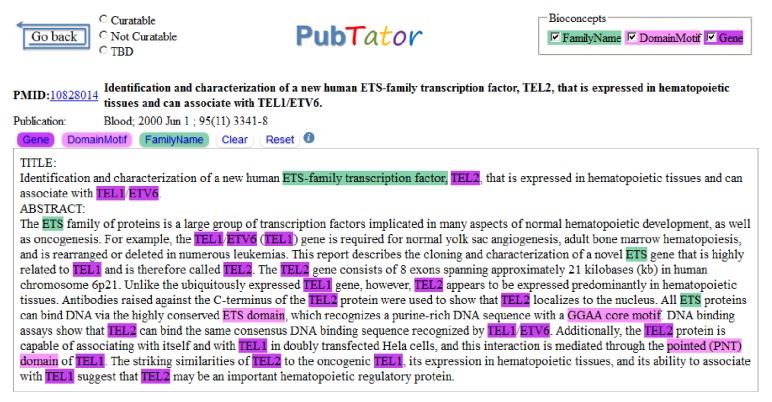
A screenshot of gene, gene family, and protein domain annotation of PMID: 10828014 in PubTator.

As a result of these challenge tasks, a number of annotated corpora were made available to the research community and have, in turn, enabled the development of a number of software tools. For instance, the BioCreative GM corpus was used to build several gene mention taggers, such as AIIA-GMT [35], BANNER [36], and BioTagger-GM [37]. However, existing gene corpora (e.g., BioCreative II GM/GN corpora [29, 30]) are annotated in either mention or document level as they were separately developed. The GM corpus (e.g., [34]) includes mention annotations but not gene identifiers of the target document; the GN corpus contains annotations for the gene identifiers but not their associated mentions. Training a supervised method on some GM data for the GN task is not ideal because different annotation criteria are often used (e.g., GM corpus may include mentions that cannot be mapped to gene identifiers). Thus, we propose developing a corpus that includes both gene mentions and concept identifiers for the same set of articles. To our best knowledge, the newly published IGN corpus [38] is the only other data set that includes both types of annotations. However, we differ from IGN in two main aspects. First, our newly developed corpus consists of more articles (694 versus 543). More importantly, we annotate gene-related concepts separately. That is, we distinguish gene, gene family, and protein domains and treat them as separate classes in our annotation (see Figure 1) as we believe such a distinction can help gene name disambiguation and improve performance. None of the current GM/GN corpora annotates these types separately. For instance, in the BioCreative II GM corpus, gene, protein family, protein domain, DNA, and RNA are all treated as gene mentions.

**Figure 2:**
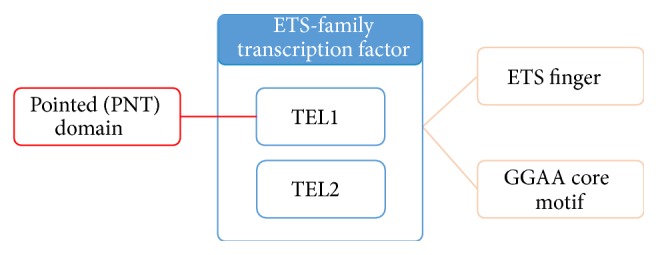
Relations between gene, gene family, and protein domains in PMID: 10828014.

Past GN systems are unable to distinguish between gene and gene families: they either completely ignored the problem or simply used a protein family name list as filters [24, 25, 39, 40]. However, the filtering strategy does not work once the family mention is not in this list. In this case, the family name becomes false positives in the results. Furthermore, detecting domain names can assist resolving ambiguous gene/protein names. As shown in Figure 2, the TEL1 and TEL2 proteins are both ETS-family transcription factors with the ETS finger domain and GGAA core motif. TEL1 also has the pointed (PNT) domain. When searching for the gene identifier in Entrez Gene, TEL1 can map to two different concepts: ATM serine/threonine kinase (gene ID: 472) and ETS translocation factor variant 6 (gene ID: 2120). But with extracted protein domain information, we can infer that in this case ETS translocation factor variant 6 is the correct answer because it is known to be associated with the PNT domain. Besides, the family name “ETS translocation factor” is also helpful to the disambiguation of TEL1/2 because it is included in the gene's official full name.

Taken together, this research makes three major contributions. First, through reannotating two existing corpora, we are the first to build a new corpus that allows the development of new methods for distinguishing different gene-related entities: (gene, gene family, and protein domains). Second, we build a new end-to-end system that includes both GM and GN modules, together with several advanced BioNLP tools (e.g., GenNorm [19], SimConcept [41], SR4GN [42], and Ab3P [43]) for improved performance. Lastly, we show state-of-the-art performance on two separate benchmark data sets.

## 2. Materials and Methods

### 2.1. Corpus Development

We reannotated two existing gene corpora. The BioCreative II GN corpus is a widely used data set for benchmarking GN tools and includes document level annotations for a total of 543 articles (281 in its training set and 262 in test). The Citation GIA test collection was recently created for gene indexing at the NLM and includes 151 PubMed abstracts with both mention level and document level annotations. They are selected because both have a focus on human genes. For both corpora, we added annotations of gene families and protein domains. For the BioCreative GN corpus, we also added mention level gene annotations. As a result, in our new corpus, there are a total of 694 PubMed articles (see Table 1). PubTator [44, 45], a tool developed and evaluated through the BioCreative III Interactive Task [46], was used as our annotation software.

### 2.2. Method Overview

**Figure 3:**
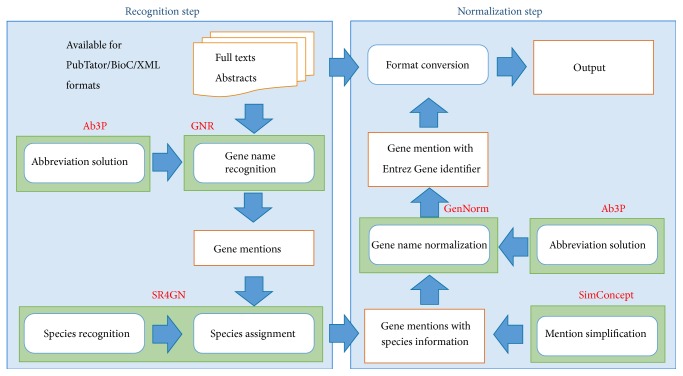
The overview of our integration method (GNormPlus).

As shown in Figure 3, our proposed approach includes two main steps: mention recognition and concept normalization, respectively. In the mention recognition step, we developed a new module, together with our previous species recognition system (i.e., SR4GN) to recognize gene and species names and match them accordingly. In concept normalization step, we applied our previous system, GenNorm, combined with a composite mention simplification tool (i.e., SimConcept) and an abbreviation resolution tool (i.e., Ab3P) for optimized performance.

### 2.3. Mention Recognition Step

In this study, we propose a supervised approach to detect the mentions of gene, gene family, and protein domain from a target input (e.g., PubMed abstracts). We first translate this mention recognition problem as a sequence labeling task. Accordingly, we adapted a probability based sequence detection conditional random fields (CRF) model [47] provided by CRF++ (http://crfpp.googlecode.com/svn/trunk/doc/index.html) library by order 2 model. CRF++ applies L-BFGS [48] which is a Quasi-Newton algorithm for large scale numerical optimization problems. We chose BIEO (B: begin, I: inside, E: end, and O: outside) label set for this recognition model. We also used the tokenization module in our previous NER systems (i.e., tmChem [28] and tmVar [15]) here. More specifically, we applied tmVar's tokenization module which splits tokens not only at punctuation (e.g., “.,()+”) and spaces, but also at digits and transitions between uppercase and lowercase. For instance, “hTIF1” will be split into three individual tokens “h,” “TIF,” and “1.” We also reused the features in tmChem and tmVar as described below.

1. *General Linguistic Features*. We included the original tokens (e.g., genes), stemmed tokens (e.g., gene), and POS tagging result (e.g., “NN”). We also extracted the prefixes and suffixes as features (length: 1~5).
2. *Character Features*. Since many gene concepts include letters, digits, and special characters, we therefore detected the number of uppercases, lowercases, letters, digits, and special characters (“;:,.->+_”).
3. *Semantic Features*. We defined three types of features to recognize the difference between potential gene mentions and other concepts. We first use the gene vocabulary from ctdbase.org (http://ctdbase.org/downloads/#allgenes) to detect those strings which can match gene mentions. In general, literature usually uses abbreviation to describe bioconcepts. We therefore use Ab3P [43] to detect those abbreviation pairs. To help the CRF model to recognize the difference between bioconcepts (e.g., genes, disease, and chemical), we collected a list of semantic tokens for genes (e.g., strains), disease (e.g., “disorder”), chemical (e.g., “trivial ring”), domain (e.g., “region”), cell (e.g., “cell”), protein symbol (e.g., glutamine), and so forth.
4. *Case Pattern Features*. We applied the case pattern features from tmVar [15]. Each token is represented in four simplified forms. Uppercase alphabetic characters are replaced by “A” and lowercase characters are replaced by “a.” Likewise, digits (0–9) are replaced by “0.” Moreover, we also merged consecutive letters and numbers and generated additional single letter “a” and number “0” as features.
5. *Contextual Features*. In order to take advantage of contextual information, for a given token we included the dictionary and linguistic features of 3 neighboring tokens from each side.

To best distinguish the three gene-related mention types, gene versus gene family versus protein domains, we applied several postprocessing rules to the CRF results. (1) Set the type by suffix (e.g., “OSBP-related proteins” to family, “LIM1 domain” to domain). (2) If we find a mention (e.g., “TIF1”) which is also a prefix of another mention (e.g., “TIF1alpha”), then we set the type of the mention to be gene family. (3) When abbreviation pairs are found, use the mention type of the long form to the sort form (e.g., “TIF1” is tagged as protein family because of its long form “transcriptional intermediary factor 1 family”). (4) If a mention occurs multiple times in an article but is tagged with different types by the CRF module, we then apply the majority rule to determine its final type in the article. For example, if hif1 was tagged twice by the CRF as a gene but as gene family in three times, then all five occurrences of hif1 will be tagged as gene family names.

### 2.4. Concept Normalization Step

The second step of our system is to map gene mentions to specific concepts in Entrez Gene. To do that, we first applied our previous GN tool, GenNorm [19, 49], which is based on a statistical inference network model via two individual matching strategies (i.e., exact match and bag-of-words match). More specifically, the exact match strategy requires the input mention to be identical to the names in the controlled vocabulary. On the other hand, the bag-of-words approach matches tokens in both input text and target vocabulary. GenNorm achieved the best performance in the BioCreative III GN task [29].

For performance optimization, we also integrated an abbreviation resolution and composite mention simplification tool in this step. First, we applied Ab3P [43] to extract the long form and short form abbreviation pairs. When the short form and long form map to different gene candidates, we typically infer the candidate gene of long form to short form for improved performance. SimConcept [41] was used to identify and resolve composite named entities, where a single span refers to more than one concept (e.g., BRCA1/2). Most past NER studies have either ignored this issue, used simple ad hoc rules, or only handled coordination ellipsis, which is only one of the many types of composite mentions studied in this work. SimConcept was shown to successfully tag individual entities from composite mentions.

## 3. Evaluation and Results

The first evaluation is a species-specific experiment where only human genes are considered. In this evaluation, we trained our system using both BioCreative II GN training set and NLM Citation GIA test collection and tested it on the BioCreative II GN test set. As shown in Table 2, we compared GNormPlus with several previously reported systems, including our previous system, GenNorm [19]. The default setting of GenNorm uses AIIA-GMT [35] for gene mention recognition. AIIA-GMT is one of the high-performing gene mention recognition tools and provided web API service. Unfortunately, AIIA-GMT is no longer available since 2013.

In the second experiment (see Table 3), we evaluate GNormPlus in multispecies gene normalization using the BioCreative III GN task data set. In this evaluation, we used the whole set of 694 articles for system training. As can be seen, our proposed method significantly outperforms previously published results in both standard *F*-measure and the task-specific TAP-k measures. The new system also outperforms our previous GenNorm tool by a significant margin.

## 4. Discussion and Conclusion

To assess the impact of using multiple gene-related mention types (i.e., gene versus family versus domain), we built a baseline model where all three types were treated as one. As shown in Table 4, the proposed multitype scheme significantly boosted the final GN performance as shown in this comparison.

Despite our best efforts, errors remain in our tagging results. Based on our results on the BioCreative II GN test set, we performed an error analysis including 127 false positive (FP) errors and 87 false negatives. In order to better understand the causes of different errors, we first separated the 214 errors by the GM step and GN step where the former accounts for 53% and the latter 47%. Among the errors in the GM step, many are due to gene/family/domain mention type confusion (e.g., assigning gene mentions to family/domain or assigning family/domain mentions to genes). Some gene mentions (e.g., TGF-beta) are particularly confusing when they refer to genes in some articles but to family/domain in other articles. In the GN step, failure in disambiguation is a frequent error (17.3%). A number of gene mentions can be associated with multiple identifiers. With only limited information in the abstract, sometimes it is very difficult to disambiguate and assign genes with correct identifiers. Another 8.9% of the errors are due to deficiencies of the gene name dictionary. Overall, as can be seen in Table 5, both the GM and GN results are important to the final performance.

To conclude, we developed GNormPlus: an end-to-end gene recognition system which handles both GM and GN tasks. By integrating several advanced BioNLP tools (i.e., GenNorm, SR4GN, Ab3P, and SimConcept), GNormPlus achieved competitive results in our two benchmarking experiments when compared with the state of the art. Unlike our previous GenNorm system that relies on AIIA-GMT, GNormPlus is a stand-alone open source tool with no dependence on external tools (freely available at http://www.ncbi.nlm.nih.gov/CBBresearch/Lu/Demo/tmTools/#GNormPlus). GNormPlus is made interoperable with other BioC-compatible BioNLP tools. For convenience, we have also applied GNormPlus to PubMed and stored its results in PubTator (http://www.ncbi.nlm.nih.gov/CBBresearch/Lu/Demo/PubTator/) so that users can readily access gene data via PubTator. In the future, we plan to explore its applications in real-world uses such as biocuration [50] and also investigate the automatic recognition of other gene-related biological entities such as microRNAs [51].

## Figures and Tables

**Table 1:** The statistic of our gene corpus.

Data set	Articles	Gene mentions (gene/family/domains)	Gene identifiers
BioCreative II GN training set	281	3,019/1,115/278	758
BioCreative II GN test set	262	3,233/1,252/361	928
NLM Citation GIA test collection	151	1,205/160/17	310

Total	694	7,457/2,527/656	1996

**Table 2:** The evaluation of human species gene normalization.

Methods	Precision	Recall	*F*-measure	System availability
Our approach (GNormPlus)	87.1%	86.4%	86.7%	Open source
GenNorm [19] + AIIA-GMT [35]	78.9%	81.4%	80.1%	GenNorm is open source but AIIA-GMT is no longer available
GNAT [23]	90.7%	82.4%	86.4%	Open source
GeNO [24]	87.8%	85.0%	86.4%	N/A
Hu et al., 2012 [40]	83.5%	82.5%	83.0%	N/A
Li et al., 2013 [39]	88.1%	92.3%	90.1%	N/A

**Table 3:** The evaluation of cross species gene normalization.

Methods	TAP-5	TAP-10	TAP-20	*F*-measure	System availability
Our approach (GNormPlus)	33.3%	36.7%	36.7%	50.1%	Open source
GenNorm [42] + AIIA-GMT [23]	32.8%	35.5%	35.5%	46.9%	GenNorm is open source but AIIA-GMT is no longer available
GeneTuKit [22]	29.7%	31.4%	32.5%	—	Open source
Kuo et al. [21]	21.4%	25.1%	25.1%	30.6%	N/A
Tsai et al. [20]	19.0%	22.9%	23.9%	—	N/A

**Table 4:** The comparison of different mention recognition training corpus.

Gene mention type scheme	Precision	Recall	*F*-measure
Gene/family/domain	87.1%	86.4%	86.7%
Single gene type only	78.4%	79.2%	78.8%

**Table 5:** The frequency of false negative and positive errors of GNormPlus.

	FN	FP	Total	Percentage
Gene mention (GM) recognition				
Gene/family/domain mention type confusion	38	18	56	27.1%
Wrong boundary or missed gene mention	18	18	36	17.4%
Not a gene mention	0	15	15	7.3%
Gene normalization (GN)				
Wrong gene identifier due to ambiguity	19	18	37	17.9%
Insufficiency of the gene name dictionary	19	0	19	9.2%
Not annotated in the gold standard	0	17	17	8.2%
Nonhuman genes found	0	11	11	5.3%
Others	13	3	16	7.7%
